# RETRACTED: Zoromba et al. Polymeric Solar Cell with 19.69% Efficiency Based on Poly(o-phenylene diamine)/TiO_2_ Composites. *Polymers* 2023, *15*, 1111

**DOI:** 10.3390/polym17141971

**Published:** 2025-07-18

**Authors:** M. Sh. Zoromba, M. H. Abdel-Aziz, A. R. Ghazy, N. Salah, A. F. Al-Hossainy

**Affiliations:** 1 Chemical and Materials Engineering Department, King Abdulaziz University, Rabigh 21911, Saudi Arabia; 2 Chemical Engineering Department, Faculty of Engineering, Alexandria University, Alexandria 21544, Egypt; 3 Physics Department, Faculty of Science, Tanta University, Tanta 31527, Egypt; 4 Center of Nanotechnology, King Abdulaziz University, Jeddah 21589, Saudi Arabia; 5 Chemistry Department, Faculty of Science, New Valley University, Al-Kharga 72511, Egypt

The journal retracts the article “Polymeric Solar Cell with 19.69% Efficiency Based on Poly(o-phenylene diamine)/TiO_2_ Composites” [1] cited above.

Following publication, concerns were brought to the attention of the publisher regarding a partial overlap of images presented in this article [1].

Adhering to our standard procedure, the Editorial Office and Editorial Board conducted an investigation that confirmed the overlap of SEM micrograph images between Figure 4a published in [1] and Figure 2b published in another paper [2], showing different compounds. Additionally, partial duplication was confirmed within Figure 4b,c.

While the authors cooperated with the Editorial Office during the investigation, they were unable to provide a satisfactory explanation for the image overlaps.

As a result, the Editorial Board has lost confidence in the reliability of the findings and has decided to retract this publication [1], as per MDPI’s retraction policy (https://www.mdpi.com/ethics#_bookmark30), accessed on 19 September 2024.

This retraction was approved by the Editor-in-Chief of the journal *Polymers*.

The authors did not agree to this retraction.
